# Non-selective beta blocker use is associated with improved short-term survival in patients with cirrhosis referred for liver transplantation

**DOI:** 10.1186/s12876-019-1155-1

**Published:** 2020-01-06

**Authors:** Taiwo Ngwa, Eric Orman, Eduardo Vilar Gomez, Raj Vuppalanchi, Chandrashekhar Kubal, Naga Chalasani, Marwan Ghabril

**Affiliations:** 1 Division of Gastroenterology and Hepatology, Indiana University School of Medicine, 702 Rotary Circle, Suite 225, Indianapolis, IN 46202 USA; 2 Transplant Surgery, Indiana University School of Medicine, Indianapolis, IN USA

**Keywords:** Beta-blockers, Cirrhosis, Liver transplantation, Mortality, Acute kidney injury

## Abstract

**Background:**

Recent evidence cautions against the use of non-selective beta-blockers (NSBB) in patients with refractory ascites or spontaneous bacterial peritonitis while other data suggests a survival benefit in patients with advanced liver disease. The aim of this study was to describe the use and impact of NSBB in patients with cirrhosis referred for liver transplantation.

**Methods:**

A single-center cohort of patients with cirrhosis, who were referred and evaluated for liver transplantation between January and June 2012 were studied for baseline characteristics and clinical outcomes. Patients were grouped according to the use of NSBB at initial evaluation, with the endpoint of 90-day mortality.

**Results:**

Sixty-five (38%) of 170 consecutive patients evaluated for liver transplantation were taking NSBB. Patients taking NSBB had higher MELD and Child Pugh score. NSBB use was associated with lower 90-day mortality (6% vs. 15%) with a risk adjusted hazard ratio of 0.27 (95%CI .09–0.88, *p* = .03). Patients taking NSBB developed acute kidney injury (AKI) within 90 days more frequently than patients not taking NSBB (22% vs 11%), *p* = 0.048). However, this was related to increased stage 1 AKI episodes, all of which resolved. Twelve (27%) of 45 patients with > 90 day follow up discontinued NSBB, most commonly for hypotension and AKI, had increased subsequent MELD and mortality.

**Conclusions:**

NSBB use in patients with cirrhosis undergoing liver transplant evaluation is associated with better short-term survival. Nevertheless, ongoing tolerance of NSBB in this population is dynamic and may select a subset of patients with better hemodynamic reserve.

## Background

Non-selective beta-blockers (NSBB) play an important role in primary and secondary prevention of variceal hemorrhage and are among the most widely used drugs in patients with cirrhosis [1–3]. They have been shown to reduce the hepatic venous portal pressure gradient by decreasing cardiac output and by inducing splanchnic vasoconstriction mediated by beta-1 and beta-2 blockade, respectively [4]. In addition, hepatic venous pressure gradient response to NSBB has been shown to reduce the risk of developing ascites, refractory ascites, and hepatorenal syndrome (HRS) [5, 6]. Independent of hemodynamic response, NSBB treatment has also been shown to decrease intestinal permeability and bacterial translocation, and to prevent the development of spontaneous bacterial peritonitis (SBP), a common and ominous infection in patients with decompensated cirrhosis [7–9].

Despite the important role of non-selective beta-blockers (NSBB) in primary and secondary prevention of variceal hemorrhage, recent evidence has cautioned against the use of NSBB in subsets of patients with decompensated cirrhosis. The use of NSBB in patients with cirrhosis with refractory ascites was associated with increased mortality in one study [10], and hemodynamic compromise, duration of hospitalization, and risk of acute kidney injury (AKI) in patients with SBP [11]. Supporting these concerns is the window hypothesis which suggests that there is a therapeutic window for NSBB treatment, which closes late in the natural progression of cirrhosis when a decreased cardiac compensatory reserve is exacerbated by NSBB with worsening outcomes [12, 13].

Recent reports however suggest that the use of NSBB is not uniformly associated with increased mortality in patients with cirrhosis and refractory ascites [14]. In a study of patients listed for liver transplantation (LT), NSBB use was associated with improved survival over a median follow up of 72 days despite the presence of refractory ascites in over 35% of patients [15]. Additionally, in a nested control study of patients listed for LT, NSBB use was associated with improved survival in patients without ascites, but increased AKI (stage≥2) in those with ascites [16].

Studies examining the use of NSBB in patients with decompensated cirrhosis are confounded by heterogeneity in patient selection, incomplete clinical characterization, and variability in clinical follow up. Furthermore, discontinuation of NSBB is not unexpected in patients with advanced cirrhosis, but its clinical correlates have not been characterized. The balance of risk and benefit of NSBB in patients with decompensated cirrhosis continues to garner contention, and the question of continuing or stopping NSBB therapy in patients referred for LT is clinically challenging. The aims of this study were to characterize the use of NSBB in patients with cirrhosis referred for LT, and to describe the impact of NSBB use on short-term mortality and AKI.

## Methods

We studied consecutive patients with cirrhosis, 18 years or older, who were referred and evaluated for LT at Indiana University between January and June 2012. The Institutional Review Board at Indiana University School of Medicine approved this study. Patients were identified from a prospectively collected transplant database. Patients with indications for LT other than cirrhosis were excluded from the study. Patients were grouped according to NSBB use at the time of initial LT evaluation, as prescribed by their referring physicians. Patients were followed until death, liver transplantation or last contact in 2015. The primary endpoint of the study was 90-day mortality from the time of initial evaluation, and the secondary outcome was 90-day AKI. The causes of death and precipitants of AKI were determined. Other events were also described including; gastrointestinal bleeding, SBP and LT. We observed that a significant proportion of patients subsequently stopped using NSBB during follow-up. We characterized patients based on continued NSBB use or discontinuation at a subsequent clinical assessment, at approximately 3 to 4-month intervals, and described the associated outcomes.

### Study parameters

The following demographic and clinical variables were recorded: age, gender, race, body mass index in kg/m^2^, heart rate in beats/min, systolic blood pressure, diastolic blood pressure, etiology of cirrhosis, presence of comorbidities (diabetes mellitus, hypertension, chronic kidney disease), laboratory data (serum bilirubin, albumin, international normalized ratio, creatinine, serum sodium), Child-Pugh score (CPS), Model for End-Stage Liver Disease (MELD), presence of and previous bleeding esophageal or gastric varices, presence and severity of ascites and hepatic encephalopathy, transjugular intrahepatic porto-systemic shunt, other complications of cirrhosis (SBP, hepatorenal syndrome (HRS), hepatopulmonary syndrome, portopulmonary hypertension, hepatocellular carcinoma), and AKI incidence and staging as recently defined by the International Ascites Club [17]. Because evidence of AKI prior to the initial LT evaluation was less well documented in referral records, we captured this event from more readily available hospitalization records. Refractory ascites was defined as patients requiring multiple therapeutic paracentesis despite diuretic therapy in the preceding 12 weeks.

### Statistical analysis

Statistical analyses were performed using Stata SE15, (Statacorp LLC., Austin, TX). Continuous variables were reported as median and inter-quartile range (IQR), and categorical variables were reported as number and percentages. Transplant-free survival analysis was performed using the Kaplan-Meier method. Univariate and multivariate Competing Risk Regression analyses were performed to determine predictors of 90-day mortality and Cox Proportional Hazard Regression analysis for the development of AKI. Post-hoc analysis included propensity score matching for analysis of risk associated with NSBB and mortality. We also reported 1-year rates of mortality and AKI for descriptive purposes.

Due to the potential for NSBB to lower mean arterial pressure (MAP), MAP was analyzed as a covariate in risk modeling. The association of MAP with 90-day mortality and AKI was also analyzed using the area under the receiver operator characteristic curve and we tested the sensitivity and specificity of a MAP threshold of < 82 mmHg, as described by Llach et al. [18], for predicting mortality and AKI. A *p* value < 0.05 was considered statistically significant for all analyses.

## Results

Of 170 consecutive patients evaluated for liver transplantation during the study period, 65 (38%) were using NSBB at the time of initial evaluation, including 36 on propranolol, 19 on nadolol and 10 on carvedilol (Additional file 1: Table S1). Patients taking NSBB had significantly higher MELD (driven by higher INR and bilirubin), CPS, Child Pugh class, and more frequent large or previously bleeding esophageal varices (Table 1). Indications for NSBB included; large or previously bleeding esophageal, gastric or ectopic varices in 42 patients, small varices with Child B or C in 15, cardiac in 2 patients and undetermined in 6 patients.

**Table 1:** Comparison of patient characteristics between those taking and not taking NSBB at the initial visit for liver transplant evaluation. Values are shown as median (interquartile range) or number (percentage)

	On NSBB *n* = 65	Not on NSBB *n* = 105	*P* value
Age, years	58 (52–64)	59 (53–63)	NS
BMI	28 (25–35)	29 (25–35)	NS
Male gender	43 (66%)	64 (64%)	NS
Diabetes mellitus	21 (32%)	33 (32%)	NS
Hypertension	28 (43%)	52 (50%)	NS
Chronic kidney disease	16 (25%)	11 (11%)	.02
Heart rate beats/min	66 (60–73)	78 (70–88)	<.001
Mean arterial pressure, mmHg	82 (76–93)	86 (78–93)	NS
Race			
Caucasian	57 (88%)	91 (87%)	NS
Black	3 (5%)	7 (7%)	
Hispanic	4 (6%)	5 (5%)	
Other	1 (1%)	2 (2%)	
Cirrhosis Etiology			
Hepatitis C	27 (42%)	57 (54%)	NS
Alcohol	23 (34%)	31 (30%)	NS
NASH	20 (30%)	22 (21%)	NS
Child Pugh Score	10 (9–11)	9 (7–10)	.001
Child Pugh class			
A	3 (5%)	16 (15%)	.01
B	24 (37%)	50 (48%)	
C	38 (58%)	39 (38%)	
MELD	16 (14–19)	14 (10–19)	0.03
INR	1.5 (1.3–1.7)	1.3 (1.2–1.6)	.04
Bilirubin mg/dL	2.7 (2–3.7)	1.9 (1.3–3.5)	.04
Creatinine mg/dL	1.1 (0.8–1.4)	0.9 (0.7–1.2)	.1
Albumin g/dL	2.9 (2.5–3.1)	3 (2.5–3.4)	NS
Sodium	135 (132–137)	135 (131–138)	NS
Esophageal varices			
None or small	24 (37%)	70 (71%)	<.001
Non-bleeding large	21 (32%)	19 (19%)	
Prior bleeding	20 (31%)	13 (13%)	
Gastric varices			
None or small	61 (94%)	100 (98%)	NS
Non-bleeding large	2 (3%)	None	
Prior bleeding	2 (3%)	2 (2%)	
Ascites			
None	12 (18%)	30 (30%)	NS
Controlled	35 (55%)	53 (51%)	
Refractory	18 (27%)	21 (20%)	
Hepatic encephalopathy			
None	18 (28%)	41 (40%)	NS
Controlled	32 (48%)	48 (47%)	
Refractory	15 (24%)	14 (13%)	
Transjugular Intrahepatic Portosystemic shunt	5 (8%)	13 (13%)	NS
Cirrhosis complications			
Spontaneous bacterial peritonitis	2 (3%)	3 (3%)	NS
Hepatorenal syndrome	2 (3%)	3 (3%)	NS
Hepatopulmonary syndrome	None	2 (2%)	NS
Portopulmonary hypertension	2 (3%)	5 (5%)	NS
Hepatocellular carcinoma	10 (15%)	26 (26%)	NS
Acute kidney injury associated with hospitalization prior to liver transplant evaluation	7 (11%)	13 (13%)	NS
NSBB indication			
Large or previously bleeding varices	42 (65%)	34 (32%)	<.001
NSBB contraindication (refractory ascites or spontaneous bacterial peritonitis)	20 (31%)	24 (23%)	NS

*Abbreviations*: *BP* blood pressure, *MELD* model for end-stage liver disease, *NSBB* non-selective beta blockers, *NS* not significant

**Fig. 1:**
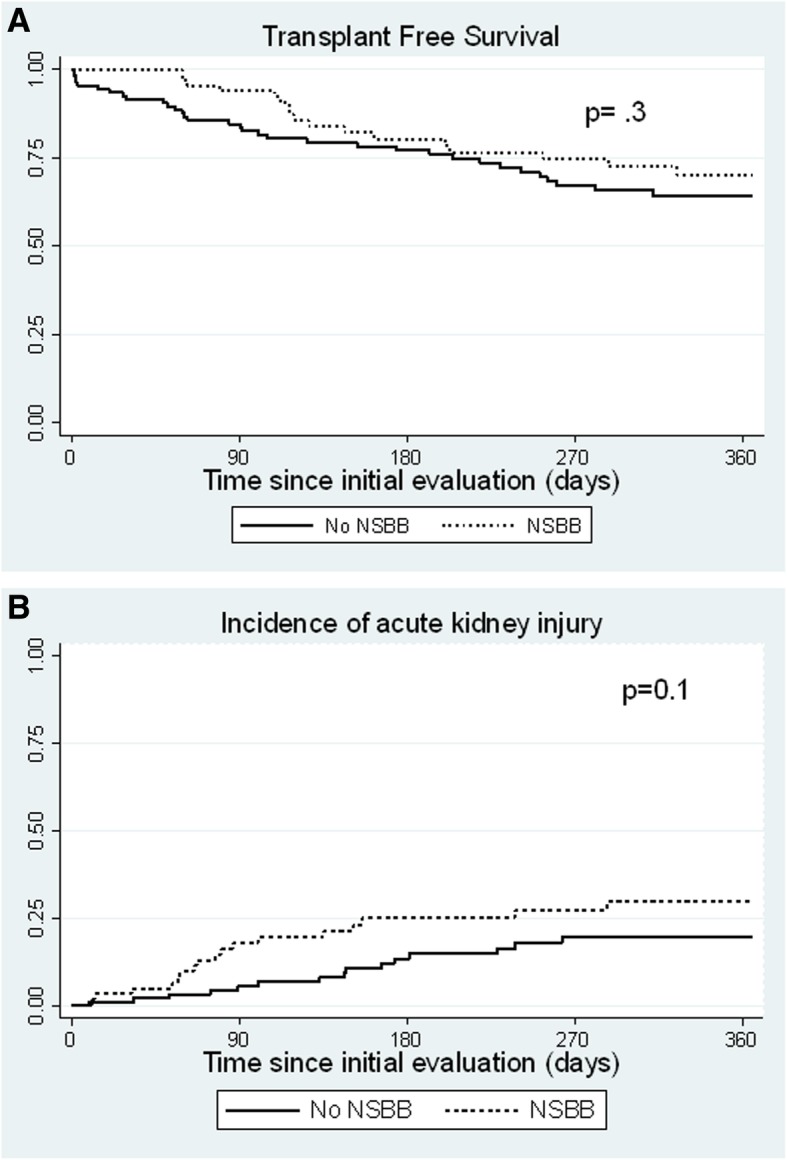
**a** Kaplan-Meier survival curves comparing transplant free survival in patients taking and not taking non-selective beta blocker (NSBB) at the time of initial liver transplant evaluation (analysis restricted to 365 days after initial evaluation). **b** Kaplan-Meier survival curves comparing the incidence of acute kidney injury during follow up in patients taking and not taking non-selective beta blocker (NSBB) (analysis restricted to 365 days after initial evaluation)

The presence of refractory ascites, SBP and prior hospitalizations for AKI were similar in patients taking or not taking NSBB. Although resting heart rate was lower in patients taking NSBB, MAP was not significantly lower. Ninety-day and overall outcomes from the date of the initial visit for liver transplant evaluation were compared in patients taking and not taking NSBB (Table 2). There were no differences in LT candidacy or transplant rates. Patients taking NSBB had more frequent episodes of AKI (22% vs. 11%, *p* = 0.048), attributed to a higher frequency of stage 1 AKI in patients taking NSBB (*p* = 0.05). There was a trend towards more frequent hospitalizations and SBP, in patients taking NSBB, but the 90-day rates of these events were similar. Although there was no significant difference in one-year transplant free survival, with median follow-up of 282 days (interquartile range 11, 843 days) (Fig. 1a), patients taking NSBB had a trend towards lower 90-day mortality (6% vs. 15%, *p* = 0.06).

**Table 2:** Clinical outcomes following initial visit for liver transplant evaluation. Values are shown as median (interquartile range) or number (percentage) unless otherwise described

	On NSBB *n* = 65	Not on NSBB *n* = 105	*P* value
Liver transplant selection committee discussion	40 (62%)	56 (53%)	
List	26 (40%)	34 (32%)	NS
Undetermined	7 (11%)	10 (10%)	
Denied	7 (11%)	12 (19%)	
Duration of follow up (months)	31 (8–64)	23.4 (6–63)	NS
Hospitalized within 90 days	18 (28%)	23 (23%)	NS
Number of hospitalizations	1 (0–3) (mean 2.2 ± 2.6)	1 (0–2) (mean 1.3 ± 1.6)	.06
Acute kidney injury			
Overall	28 (43%)	22 (20%)	.002
Stage 1	14 (50%)	2 (10%)	.009
Stage2	5 (18%)	7 (32%)	
Stage 3	9 (32%)	13 (59%)	
90-day	14 (22%)	11 (11%)	.048
Stage 1	5 (36%)^b^	1 (9%)^b^	NS
Stage2	3 (21%)	4 (36%)	
Stage 3	6 (43%)	6 (55%)	
Gastrointestinal bleeding^a^			
Overall	10 (15%)	9 (9%)	NS
90-day	None	None	
Spontaneous bacterial peritonitis			
Overall	9 (14%)	6 (6%)	0.09
90-day	4 (6%)	2 (2%)	NS
Liver transplant			
Overall	21 (33%)	32 (31%)	NS
90-day	1 (2%)	5 (5%)	
Mortality			
Overall	32 (49%)	45 (43%)	NS
90-day	4 (6%)	16 (15%)	.06

Values shown as median (interquartile range) or number (percentage) unless otherwise specified

*Abbreviations*: *NSBB* non-selective beta blockers, *NS* not significant

^a^Related to portal hypertension ^b^for the comparison of stage 1 AKI *p* = .05

Forty-five patients initially taking NSBB had subsequent clinical evaluations at median intervals of 113 days (IQR 93–141) from baseline. These patients were examined for descriptive purposes only given the small sample size. Discontinuation of NSBB during that time was associated with increased subsequent MELD and a trend for increased 90-day mortality (Table 3). Twelve (27%) patients overall discontinued NSBB, including 6 during hospitalization (for hypotension and AKI in 5, and unknown reasons in 1), and 6 as outpatients (for hypotension in 3 and unknown reasons in 3). Patients who discontinued NSBB had more frequent chronic kidney disease, but less frequent diabetes mellitus and hypertension, and lower MAP at baseline (Additional file 1: Table S2). More patients on propranolol discontinued NSBB during follow up. A comparison of baseline characteristics based on type of NSBB used was notable for greater proportion of Child class C in patients on propranolol and nadolol, and lower MAP (not reaching statistical significance) in patients on propranolol (Additional file 1: Table S1).

**Table 3:** Comparison of characteristics and outcomes of patients initially taking NSBBs and continuing or discontinuing NSBBs at subsequent time points following initial visit for liver transplant evaluation. Values shown as median (interquartile range) or number (percentage). Patients taking stopping NSBB by a median interval of 113 days are compared for clinical parameters at that time and outcomes in the subsequent 90 days or until the next clinical evaluation. Patients subsequently stopping NSBB before evaluation at a median interval of 238 days from baseline are compared for clinical parameters at that time and outcomes in the subsequent 90 days

Clinical characteristics	Baseline evaluation	First clinical re-evaluation Median 113 days from baseline *n* = 45		Second clinical re-evaluation Median 238 days from baseline *n* = 21	
	NSBB *N* = 65	On NSBB *n* = 34	Not on NSBB *n* = 9	On NSBB *n* = 18	Not on NSBB *n* = 3
Mean arterial pressure, mmHg	82 (76–93)	89 (75–96)	79 (73–84)	85 (79–96)	87 (69–95)
MELD	16 (14–19)	**15 (12–19)	**23 (17–27)	14 (12–17)	14 (11–21)
Creatinine (mg/dL)	1.1 (0.8–1.4)	*1 (0.8–1.3)	*1.8 (1–2.2)	0.9 (0.7–1.3)	0.8 (0.7–1.5)
Child Pugh Score	10 (9–11)	10 (8–11)	11 (10–12)	9 (8–10)	8 (7–12)
NSBB at baseline					
Propranolol	36 (56%)	**15 (45%)	**9 (100%)	7 (35%)	2 (67%)
Nadolol	19 (29%)	12 (35%)	None	9 (53%)	None
Carvedilol	10 (15%)	7 (20%)	None	2 (12%)	1 (33%)
Clinical events occurring *after* the specified evaluation					
Acute kidney injury	14 (21%)	3 (9%)	None	**None	**1 (5%)
Gastrointestinal bleeding^a^	None	None	None	None	None
Spontaneous bacterial peritonitis	4 (6%)	None	None	1 (5%)	None
Liver transplant	1 (2%)	6 (18%)	1 (11%)	None	None
Mortality	4 (8%)	*1 (3%)	*2 (22%)	1 (5%)	None
Lost to follow up	None	None	None	2 (12%)	1 (13%)

*Abbreviations*: *MELD* model for end-stage liver disease, *NSBB* non-selective beta blockers

^a^Related to portal hypertension

*p < 0.1 for comparison

**p < 0.05 for comparison

The cause of death could be ascertained in 21 patients who died within 90 days of initial evaluation and was liver related in all 4 patients on NSBB and in 10 of 17 patients not taking NSBB (*p* = .2) (Additional file 1: Table S3). The predictors of all-cause 90-day mortality are summarized in Table 4. The use of NSBB was independently associated with lower 90-day mortality, as were lower MAP, MELD, and interval AKI (stage ≥2). The association of NSBB with lower 90-day mortality persisted in modeling using propensity score matching for NSBB use at baseline (Additional file 1: Table S4).

**Table 4:** Predictors of 90-day mortality in the first 90 days on univariate and multivariate Competing Risk (liver transplant as the competing risk for mortality) analysis

	Univariate		Multivariate	
	Sub-Hazard Ratio (95% CI)	*P* value	Sub-Hazard Ratio (95% CI)	*P* value
MELD	1.2 (1.1–1.3)	<.001	1.1 (1.02–1.15)	.008
Mean arterial pressure (mmHg)	0.93 (0.89–0.97)	<.001	0.95 (0.9–0.99)	.03
NSBB use	0.35 (0.12–1.02)	.05	0.29 (.09–.95)	.04
AKI (≥stage 2) within 90 days	11 (4.7–25.6)	<.001	4.4 (1.3–15.4)	.02
Child Pugh Score	1.22 (0.99–1.5)	.05		
Gender (male)	2.6 (0.9–7.8)	.09		

Factors not predictive of 90-day mortality included; Age, body mass index, race, etiology of liver disease (hepatitis C, alcoholic or non-alcoholic fatty liver disease), serum albumin and serum sodium or prophylactic antibiotics. Stage 1 AKI was not associated with 90-day mortality and the analysis of AKI within 90-days was restricted to patients with AKI ≥ stage 2

The results of the final model did not differ when including hospitalization for acute kidney injury prior to liver transplant evaluation

*Abbreviations*: *AKI* acute kidney injury, *MELD* model for end-stage liver disease, *NSBB* non-selective beta blockers

There was a trend toward increased AKI in patients taking NSBB (Fig. 1b). Among 25 patients developing AKI within 90 days, the precipitating factor was deemed to be pre-renal in 23 (unknown or non-prerenal causes in 2 patients). Of these, AKI was related to infection in 3 (23%) of 13 patients taking NSBB and in 5 (50%) of 10 patients not taking NSBB, *p* = 0.2 (Additional file 1: Table S5). The course of AKI resulted in renal recovery in 15 of the 25 patients (6 of 6 (stage 1), 3 of 7 (stage 2), and 6 of 12 (stage 3) AKI). The median (IQR) serum creatinine levels (mg/dL) in those recovering renal function measured pre-AKI, peak-AKI and post-AKI were 1.1 (1–1.3), 2.8 (1.7–4.4) and 1.3 (1–1.6), respectively. Ten patients with AKI died (4 (stage 2) and 6 (stage 3) AKI). The predictors of AKI within 90-days are summarized in Table 5. After adjusting for MELD, CPS, and MAP, NSBB use was not associated with AKI, with MELD being the only independent predictor.

**Table 5:** Predictors of acute kidney injury within 90 days of initial liver transplant evaluation visit on univariate and multivariate Cox Proportional Hazard Regression analysis. The unadjusted hazard ratio for NSBB use and acute kidney injury between 90 and 365 days of initial evaluation was 0.97, 95% confidence interval 0.4 to 2.7, *p* = 0.9

	Univariate		Multivariate	
	Hazard Ratio (95% CI)	*P* value	Hazard Ratio (95% CI)	*P* value
MELD	1.17 (1.08–1.29)	<.001	1.19 (1.07–1.3)	.001
Mean arterial pressure (mmHg)	0.96 (.92–1.00)	.08		
NSBB	3.4 (1.2–9.8)	.02		
Child Pugh Score	1.44 (1.13–1.83)	.003		

Factors not predictive of acute kidney injury within 90 days included; NSSB use, age, gender, body mass index, race, etiology of liver disease (hepatitis C, alcoholic or non-alcoholic fatty liver disease), diabetes mellitus, hypertension and serum albumin and sodium

When analysis was performed for prediction of ≥ stage 2 acute kidney injury within 90 days, the unadjusted hazard ratio for NSBB use was 4.5, 95% CI 0.9–22, *p* = 0.06, and the adjusted hazard ratio was 0.7, 95% CI 0.08–6.3, *p* = 0.7 on (adjusted for age, MELD, mean arterial pressure and Childs Pugh score)

*Abbreviations*: *MELD* model for end-stage liver disease, *NSBB* non-selective beta blockers

For descriptive purposes we contrasted outcomes in the 44 patients with refractory ascites or a history of SBP, MELD and CPS were similar in patients taking or not taking NSBB, as were 90-day mortality and AKI (Additional file 1: Table S6).

**Fig. 2:**
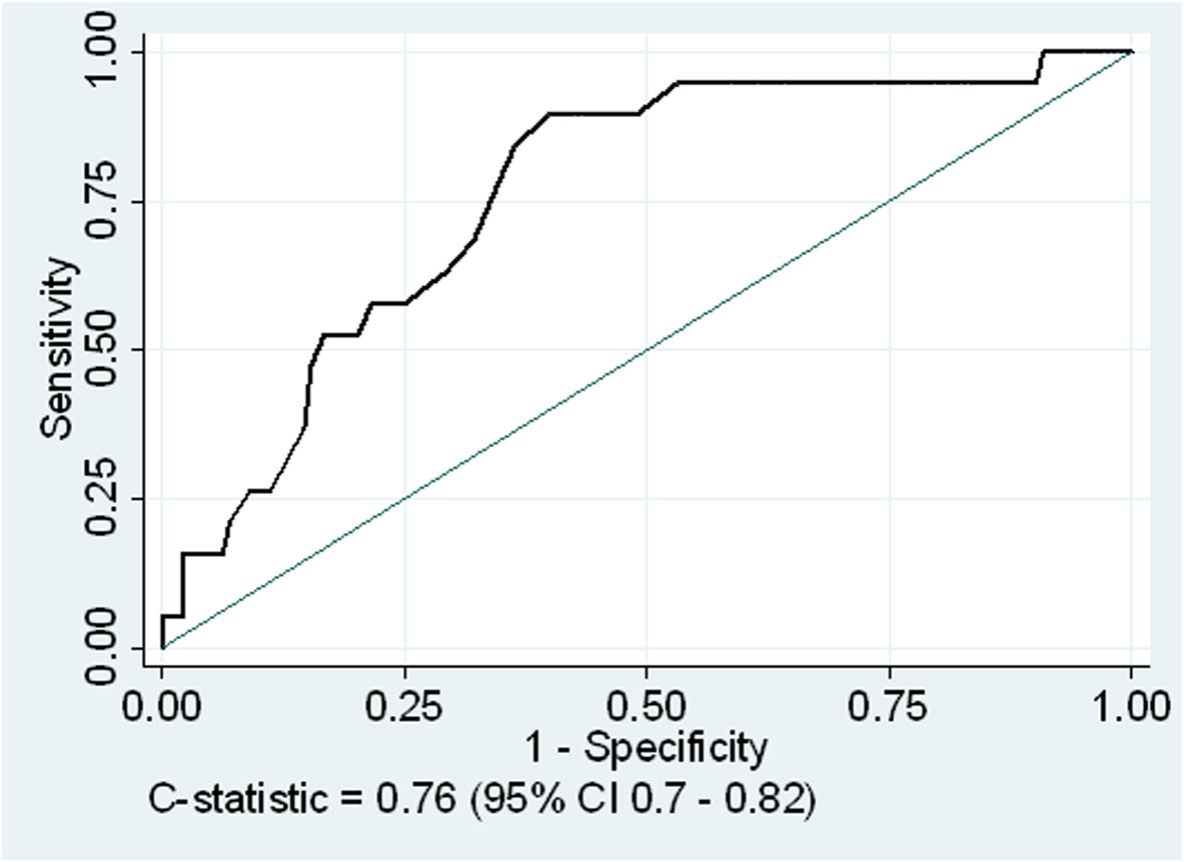
The area under the receiver operator characteristic curve for the association of mean arterial pressure at baseline with 90-day mortality

Given the expected effect of NSBB on lowering MAP (lower MAP was associated with increased 90-day mortality and AKI), we analyzed the relationship of MAP at baseline with 90-day mortality and AKI using the area under the receiver operator characteristic curve (Additional file 1: Table S7). The C-statistic for MAP and 90-day mortality in all patients was 0.76 (95% CI, 0.7 to 0.82) (Fig. 2), however the C-statistic was lower in patients taking NSBB for both mortality and AKI. A MAP < 82 mmHg was less sensitive and less specific for predicting these endpoints in patients taking NSBB. Thirteen (36%) of 36 patients not taking NSBB with a baseline MAP< 82 mmHg died within 90 days, while 3 (5%) of 63 patients with a higher MAP, *p* < 0.001. On the other hand, only 3 (9%) of 32 patients taking NSBB with a MAP< 82 mmHg died within 90 days, and 1 (3%) of 33 with a higher MAP, *p* = 0.3 (6 patients not on NSBB were missing baseline MAP values).

## Discussion

This study characterized in detail patients with cirrhosis evaluated for LT, and 38% were taking NSBB, as prescribed by their referring providers, with real-world heterogeneity of types and dosing of these agents. Although they had more advanced liver disease by MELD and Child Pugh score, they had paradoxically better short-term transplant free survival compared to patients not taking NSBB. Our observations support those from other centers describing survival benefit of NSBB use in patients listed for LT [15, 19]. The role of NSBB in end stage liver disease and the evidence for potential benefits in decreasing portal pressure, bacterial translocation and systemic inflammation are succinctly summarized in a recent review, and provide a rationale for survival benefit for NSBB use extending beyond the prevention of variceal bleeding [20]. Several factors may also contribute to this finding. Patients not taking NSBB had more frequent non-liver related deaths during follow up. Patients referred for LT may represent a select group that received closer clinical follow-up during transplant evaluation (although less than half were listed), and the relatively high transplant rates at our center may have minimized risks related to underlying hepatic or renal dysfunction.

We contrasted our NSBB using subgroup to cohorts with reported increased mortality with refractory ascites treated with propranolol [10]. Notably, NSBB treated patients in that cohort had worse baseline characteristics compared with NSBB treated patients in our cohort (refractory/intractable ascites 100% vs. 29%, Child class C, 74% vs. 58%, mean MELD 19 ± 4 vs. 17 ± 5, median serum sodium 125 vs. 135 mmol/L and median systolic blood pressure 103 vs. 112 mmHg). Additionally, that cohort’s median daily propranolol dose was 80 mg, contrasting with 20 mg in our propranolol treated patients, who also had the highest rate of NSBB discontinuation during follow up. It is important to remember that the type and dose of NSBB were determined by the referring physicians and represented the real-world clinical experience of the cohort. We suspect that patients in our study were on low doses of propranolol due to limited hemodynamic reserves, which would also explain their higher rate of NSBB discontinuation over time.

Few patients in our study had a history of SBP to meaningfully assess for increased mortality and AKI with NSBB reported by others [11]. We did not observe increased 90-day mortality or AKI in patients taking NSBB when exclusively examining patients with refractory ascites or a history of SBP. However, this was a small subgroup, and it is important to note the significant limitation of diagnosing refractory ascites retrospectively, as in our study. Hence, patients may be incorrectly categorized as having refractory ascites and the analysis may be underpowered to detect clinically important differences. This suggests that even if these contraindications to NSBB therapy are not absolute, they indicate the need for judicious assessment of NSBB tolerance.

An important limitation of our and many other studies is that NSBB use was not randomized but determined by indications for and tolerance of NSBB, which reflect severity of liver disease and hemodynamic vulnerability, respectively. Similar to a recent report, not all patients with a history of large or bleeding varices were on NSBB at the time of initial evaluation [21]. Consequently, patients tolerating NSBB despite their advanced disease may represent a self-selected sub-population with better hemodynamic reserve and arguably better prognosis. Our analysis of NSBB discontinuation, albeit in a small subset of patients, led to the main novelty and thrust of our study. We postulate that this phenomenon may explain the apparent survival advantage for NSBB use seen in our and other retrospective studies. In support of this concept is that NSBB use was not associated with lower MAP despite higher MELD and CPS. In further support of this concept, we observed discontinuation of NSBB during follow up in 27% of patients initially taking NSBB, with increased subsequent MELD (driven by renal dysfunction) and a trend for increase mortality. The lack of transplant-free survival benefit beyond 90-days with NSBB use at baseline further supports the theory that patients tolerating NSBB are self-selected in this retrospective study. Since some patients became NSBB intolerant during follow-up with worse outcomes, it is difficult to ascribe long-term benefit to NSBB use in our cohort. In other words, rather than being a driver of better survival, tolerance of NSBB, despite advanced liver disease, SBP or refractory ascites, may be a surrogate marker of better hemodynamic reserve, with that reserve being a plausible reason for better short-term prognosis. Conversely, patients with advanced liver disease and intolerance of NSBB (due to hemodynamic intolerance or AKI in our cohort) may be a surrogate marker for worse hemodynamic reserve and related short-term prognosis.

Hypotension and AKI were the main indications for stopping NSBB. Notably, NSBB treated patients in our study were on relatively low doses of the respective agents. This suggests that the evolution of liver and renal dysfunction, and hemodynamic vulnerability in these patients is an important driver of NSBB intolerance and risk of poor outcomes. These findings are consistent with those of the Italian Multicentre Project for Propanolol in Prevention of Bleeding [22] which observed discontinuation of NSBB in 26% of patients due to intolerance. Bossen et al. observed a 29% rate of NSBB discontinuation in association with increased mortality [23].

Discontinuation of NSBB in our cohort during follow up may have ameliorated potentially negative effects in vulnerable patients over time. Overall mortality rates (beyond 90 days) were similar for patients taking and not taking NSBB, a finding that predominates when considering recent analyses. A recently published meta-analysis that included 3 randomized controlled trials, and a post-hoc analysis of 3 randomized controlled trials examining NSBB therapy in patients with cirrhosis and ascites revealed similar overall survival with or without NSBB use [14, 23].

Treatment with NSBB may be detrimental to the hemodynamics of decompensated patients with cirrhosis who already have a poor circulatory reserve, predisposing them to the development of renal impairment [24–26]. This study provided detailed characterization of AKI during follow up. While AKI was more frequent in NSBB treated patients, the difference was wholly attributed to stage 1 episodes, all of which resolved. The mortality risk associated with AKI was only observed in stage ≥2 injuries. Although the risk adjusted analysis indicated no association for NSBB use with 90-day AKI (regardless of severity) we noted more frequent non-infection related pre-renal injuries in NSBB treated patients, where the hemodynamic effects of lowering blood pressure may have played a role in AKI. Hypotension and AKI were also the main reasons for NSBB discontinuation when documented, and are in line with the Baveno VI, U.K. and U.S. guidelines advising caution or discontinuation of NSBB with renal impairment or hypotension (systolic blood pressure < 90 mmHg), albeit in patients with refractory ascites [27–29].

Mean arterial pressure at initial evaluation was as independent predictor of 90-day mortality which supports previous studies associating low MAP with increased mortality in patients with cirrhosis and ascites [18, 25]. However, the C-statistic for this association was higher in patients not using NSBB, and a MAP< 82 mmHg was only discriminating for 90-day mortality in patient not taking NSBB. It is possible that low MAP on NSBB is related to the antihypertensive effect of therapy, while low MAP without NSBB is more reflective of peripheral vasodilatation and overextended compensatory mechanisms related to advanced liver disease. Hence, the absolute MAP may be less important than the true MAP (off NSBB) in reflecting an individual’s hemodynamic reserve and prognosis. These findings support the “window hypothesis” [13], but points to challenges in utilizing a MAP threshold of 82 mmHg to determine closure of the therapeutic window in patients already taking NSBB. This also further supports the hypothesis that tolerance of NSBB may serve as a surrogate marker of more stable liver disease, better hemodynamic reserve and favorable prognosis.

The limitations of the study include the retrospective nature, small sample size, heterogeneity in NSBB indication, type and dosing, missing data, and the risk of aforementioned selection bias. We were not able to elucidate the reasons for NSBB discontinuation in all cases, and MAP was based on a single blood pressure measurement. The strengths of the study include the thorough characterization of patients and clinical outcomes, including staging of AKI, and characterization of NSBB discontinuation during follow up.

## Conclusions

In conclusion, NSBB use in patients undergoing LT evaluation was associated with more advanced liver disease but better short-term survival. NSBB discontinuation was common and mainly attributed to hypotension and stage 1 AKI. The latter was reversible and not associated with increased mortality. NSBB tolerance may represent a surrogate marker of more stable liver disease. The potential interplay of competing effects of NSBB and MAP remains undefined for guiding optimal use and tolerance of therapy. This study underscores the need for close monitoring and serial assessment of NSBB tolerance, and highlights the need for prospective studies to determine the potential benefits and risks of NSBB therapy in advanced cirrhosis.

## Supplementary information

**Additional file 1: Table S1.** Comparison of selected patient characteristics based on the type of NSBB used at the time of initial evaluation. Values are shown as median (interquartile range) or number (percentage) unless otherwise noted. **Table S2.** Comparison of select patient characteristics in patient continuing and discontinuing NSBBs over 90 to 270 days after the initial evaluation. Values are shown as median (interquartile range) or number (percentage). **Table S3.** The cause of death in patients who died within 90 days of initial evaluation in patients taking and not taking NSBB. **Table S4.** The association of NSBB use with 90-mortality in models using propensity score matching for use of NSBB at baseline. **Table S5.** The precipitating factors of acute kidney injury developing within 90 days of initial evaluation. **Table S6.** Comparison of selected characteristics and 90-day outcomes of patients with refractory ascites or a history of spontaneous bacterial peritonitis prior to initial evaluation for liver transplantation grouped by NSBB use. Values are shown as median (interquartile range) or number (percentage). **Table S7.** The association of mean arterial pressure at the time of initial liver transplant evaluation with 90-day mortality and acute kidney injury using the area under the receiver operator characteristic curve, and sensitivity and specificity for a MAP<82mmHg to predict these endpoints.

## Data Availability

The datasets generated and/or analyzed during the current study are not publicly available due to confidentiality of human research data from our institution, but are available from the corresponding author on reasonable request.

## Abbreviations

AKI: Acute kidney injury
CPS: Child Pugh Score
HRS: Hepatorenal syndrome
IQR: Interquartile range
LT: Liver transplantation
MAP: Mean arterial pressure
MELD: Model for End-stage Liver Disease
NSBB: Non-selective beta blockers
SBP: Spontaneous bacterial peritonitis

## References

[1] de la Pena J (2005). Variceal ligation plus nadolol compared with ligation for prophylaxis of variceal rebleeding: a multicenter trial. Hepatology.

[2] Ge PS, Runyon BA (2014). The changing role of beta-blocker therapy in patients with cirrhosis. J Hepatol.

[3] Tripathi D (2009). Randomized controlled trial of carvedilol versus variceal band ligation for the prevention of the first variceal bleed. Hepatology.

[4] Garcia-Tsao G, Bosch J (2010). Management of varices and variceal hemorrhage in cirrhosis. N Engl J Med.

[5] Abraldes JG (2003). Hemodynamic response to pharmacological treatment of portal hypertension and long-term prognosis of cirrhosis. Hepatology.

[6] Hernandez-Gea V (2012). Development of ascites in compensated cirrhosis with severe portal hypertension treated with beta-blockers. Am J Gastroenterol.

[7] Reiberger T (2013). Non-selective betablocker therapy decreases intestinal permeability and serum levels of LBP and IL-6 in patients with cirrhosis. J Hepatol.

[8] Senzolo M (2009). Beta-blockers protect against spontaneous bacterial peritonitis in cirrhotic patients: a meta-analysis. Liver Int.

[9] Thalheimer U (2005). Infection, coagulation, and variceal bleeding in cirrhosis. Gut.

[10] Serste T (2010). Deleterious effects of beta-blockers on survival in patients with cirrhosis and refractory ascites. Hepatology.

[11] Mandorfer M (2014). Nonselective beta blockers increase risk for hepatorenal syndrome and death in patients with cirrhosis and spontaneous bacterial peritonitis. Gastroenterology.

[12] Krag A (2010). Low cardiac output predicts development of hepatorenal syndrome and survival in patients with cirrhosis and ascites. Gut.

[13] Krag A (2012). The window hypothesis: haemodynamic and non-haemodynamic effects of beta-blockers improve survival of patients with cirrhosis during a window in the disease. Gut.

[14] Chirapongsathorn S (2016). Nonselective beta-blockers and survival in patients with cirrhosis and ascites: a systematic review and meta-analysis. Clin Gastroenterol Hepatol.

[15] Leithead JA (2015). Non-selective beta-blockers are associated with improved survival in patients with ascites listed for liver transplantation. Gut.

[16] Kim SG (2017). Beneficial and harmful effects of nonselective beta blockade on acute kidney injury in liver transplant candidates. Liver Transpl.

[17] Angeli P (2015). Diagnosis and management of acute kidney injury in patients with cirrhosis: revised consensus recommendations of the International Club of Ascites. Gut.

[18] Llach J (1988). Prognostic value of arterial pressure, endogenous vasoactive systems, and renal function in cirrhotic patients admitted to the hospital for the treatment of ascites. Gastroenterology.

[19] Onali Simona, Kalafateli Maria, Majumdar Avik, Westbrook Rachel, O'Beirne James, Leandro Gioacchino, Patch David, Tsochatzis Emmanuel A. (2017). Non-selective beta-blockers are not associated with increased mortality in cirrhotic patients with ascites. Liver International.

[20] Moctezuma-Velazquez C, Kalainy S, Abraldes JG (2017). Beta-blockers in patients with advanced liver disease: has the dust settled?. Liver Transpl.

[21] Pfisterer N (2018). Betablockers do not increase efficacy of band ligation in primary prophylaxis but they improve survival in secondary prophylaxis of variceal bleeding. Aliment Pharmacol Ther.

[22] The Italian Multicenter Project for Propranolol in Prevention of Bleeding" as institutional author name and "Propranolol prevents first gastrointestinal bleeding in non-ascitic cirrhotic patients. Final report of a multicenter randomized trial. J Hepatol. 1989;9(1):75–83. 2671121

[23] Bossen L (2016). Nonselective beta-blockers do not affect mortality in cirrhosis patients with ascites: post hoc analysis of three randomized controlled trials with 1198 patients. Hepatology.

[24] Gines P (2004). Management of cirrhosis and ascites. N Engl J Med.

[25] Serste T (2011). Beta-blockers cause paracentesis-induced circulatory dysfunction in patients with cirrhosis and refractory ascites: a cross-over study. J Hepatol.

[26] Ruiz-del-Arbol L (2003). Systemic, renal, and hepatic hemodynamic derangement in cirrhotic patients with spontaneous bacterial peritonitis. Hepatology.

[27] Tripathi D (2015). U.K. guidelines on the management of variceal haemorrhage in cirrhotic patients. Gut.

[28] de Franchis R, Baveno VIF (2015). Expanding consensus in portal hypertension: report of the Baveno VI consensus workshop: stratifying risk and individualizing care for portal hypertension. J Hepatol.

[29] Garcia-Tsao G (2017). Portal hypertensive bleeding in cirrhosis: risk stratification, diagnosis, and management: 2016 practice guidance by the American association for the study of liver diseases. Hepatology.

