# Supplementary material for: Non-selective beta blocker use is associated with improved short-term survival in patients with cirrhosis referred for liver transplantation
Source: BMC Gastroenterol. 2020 Jan 6;20:4. doi: 10.1186/s12876-019-1155-1 (PMC6945622; doi:10.1186/s12876-019-1155-1)
Supplement: Supplementary file 1 — Additional file 1: Table S1. Comparison of selected patient characteristics based on the type of NSBB used at the time of initial evaluation. Values are shown as median (interquartile range) or number (percentage) unless otherwise noted. Table S2. Comparison of select patient characteristics in patient continuing and discontinuing NSBBs over 90 to 270 days after the initial evaluation. Values are shown as median (interquartile range) or number (percentage). Table S3. The cause of death in patients who died within 90 days of initial evaluation in patients taking and not taking NSBB. Table S4. The association of NSBB use with 90-mortality in models using propensity score matching for use of NSBB at baseline. Table S5. The precipitating factors of acute kidney injury developing within 90 days of initial evaluation. Table S6. Comparison of selected characteristics and 90-day outcomes of patients with refractory ascites or a history of spontaneous bacterial peritonitis prior to initial evaluation for liver transplantation grouped by NSBB use. Values are shown as median (interquartile range) or number (percentage). Table S7. The association of mean arterial pressure at the time of initial liver transplant evaluation with 90-day mortality and acute kidney injury using the area under the receiver operator characteristic curve, and sensitivity and specificity for a MAP<82mmHg to predict these endpoints. [file 12876_2019_1155_MOESM1_ESM.docx]

**Table S1** Comparison of selected patient characteristics based on the type of NSBB used at the time of initial evaluation. Values are shown as median (interquartile range) or number (percentage) unless otherwise noted.

|  | **Propranolol**  **n=36** | **Nadolol**  **n=19** | **Carvedilol**  **n=10** | **P value** |
| --- | --- | --- | --- | --- |
| Age, years | 57 (52 - 63) | 62 (56 - 66) | 59 (55 - 65) | NS |
| Male gender | 24 (67%) | 13 (68%) | 6 (60%) | .8 |
| Diabetes mellitus | 9 (25%) | 6 (32%) | 6 (60%) | .1 |
| Hypertension | 14 (39%) | 7 (37%) | 7 (70%) | NS |
| Chronic kidney disease | 11 (30%) | 2 (11%) | 3 (30%) | NS |
| Cirrhosis Etiology  Hepatitis C  Alcohol  NASH | 20 (56%)  16 (34%)  8 (30%) | 3 (16%)  6 (30%)  7 (21%) | 4 (40%)  1 (10%)  5 (50%) | .02  NS  .1 |
| Median and mean daily dose (mg per day) | 20 (20 – 40)  29±17 | 20 (20 – 40)  29±12 | 9.4 (6.25 – 12.5)  10±7 | NA |
| Heart rate beats/min | 66 (61- 75) | 65 (58 - 70) | 70 (58 - 85) | NS |
| Mean arterial pressure, mmHg | 79 (75 - 92) | 85 (78 - 93) | 90 (81 - 94) | NS |
| Child Pugh Score  Child Pugh class  A  B  C | 10 (9 - 11)  1 (3%)  10 (28%)  25 (69%) | 10 (7 - 12)  2 (11%)  7 (21%)  10 (53%) | 9 (8 - 10)  None  7 (70%)  3 (3%) | .1  .09 |
| Model for End-Stage Liver Disease | 17 (15 - 20) | 17 (13 - 22) | 14 (12 - 16) | NS |
| Albumin g/dL | 2.8 (2.3 – 3.1) | 2.9 (2.6 – 3.1) | 3.3 (3 – 3.6) | .06 |
| Esophageal varices  None or small  Non-bleeding large  Prior bleeding | 15 (42%)  10 (28%)  11 (30%) | 7 (21%)  9 (47%)  3 (16%) | 2 (20%)  2 (20%)  6(60%) | .1 |
| Gastric varices  None or small  Non-bleeding large  Prior bleeding | 34 (94%)  2 (6%)  None | 18 (95%)  None  1 (1%) | 9 (90%)  None  1 (10%) | NS |
| Acute kidney injury associated with hospitalization prior to liver transplant evaluation | 11 (31%) | 5 (26%) | 4 (40%) | NS |
| Refractory ascites or SBP prior to evaluation | 14 (39%) | 3 (16%) | 3 (30%) | NS |

Abbreviations: NA, not applicable; NSBB, non-selective beta blockers; NS, not significant; SBP, spontaneous bacterial peritonitis

* Related to portal hypertension

There were no differences in body mass index, race, Transjugular Intrahepatic Portosystemic shunt, INR, serum bilirubin, creatinine and sodium, presence or severity of ascites or hepatic encephalopathy. There were also no differences in baseline complications of liver disease (SBP, hepatorenal syndrome, hepatopulmonary syndrome, portopulmonary hypertension and hepatocellular carcinoma)

**Table S2** Comparison of select patient characteristics in patient continuing and discontinuing NSBBs over 90 to 270 days after the initial evaluation. Values are shown as median (interquartile range) or number (percentage).

|  | **Continued NSBB**  **n=33** | **Discontinued NSBB**  **n=12** | **P value** |
| --- | --- | --- | --- |
| Age, years | 59 (55 - 64) | 57 (51 - 65) | NS |
| Male gender | 24(73%) | 5 (42%) | .05 |
| Diabetes mellitus | 15(46%) | 2 (17%) | .08 |
| Hypertension | 19 (58%) | 1 (15%) | .003 |
| Chronic kidney disease | 6 (18%) | 6 (50%) | .03 |
| Type of NSBB  Propranolol  Nadolol  Carvedilol | 13 (39%)  13 (39%)  7 (21%) | 11 (92%)  None  1 (8%) | .007 |
| Heart rate beats/min | 64 (58 - 72) | 70 (62 - 80) | NS |
| Mean arterial pressure, mmHg | 86 (79 - 94) | 77 (72 - 81) | .02 |
| Child Pugh Score  Child Pugh class  A  B  C | 10 (8 - 11)  2 (6%)  13 (39%)  18 (55%) | 11 (10 - 11)  None  2 (17%)  10 (83%) | .1  NS |
| Model for End-Stage Liver Disease | 16 (14 - 18) | 16 (15 - 19) | NS |
| Creatinine mg/dL | 1.1 (0.8 – 1.3) | 1 (0.9 – 1.5) | NS |
| Sodium | 136 (133 – 137) | 133 (129 – 136) | .06 |
| Acute kidney injury associated with hospitalization prior to liver transplant evaluation | 3 (9%) | 1 (8%) | NS |
| Refractory ascites or SBP at or prior to initial evaluation | 10 (30%) | 5 (42%) | NS |
| Number of hospitalizations | 1 (1 – 3)  (mean 2 ± 1.9) | 2 (2 – 3)  (mean 4.2 ±3.6) | .04 |
| Time to hospitalization (days) | 156 (107 – 303) | 90 (32 – 133) | .04 |
| Overall outcomes  Acute kidney injury  Gastrointestinal bleeding*  SBP  Liver transplant  Died | 3 (10%)  9 (27%)  3 (10%)  15 (45%)  13 (24%) | 6 (50%)  1 (8%)  3 (25%)  4 (33%)  5 (42%) | .002  NS  NS  NS  NS |

Abbreviations: BP, blood pressure; NSBB, non-selective beta blockers; NS, not significant; SBP, spontaneous bacterial peritonitis

* Related to portal hypertension

There were no differences in body mass index, race, etiology of liver disease, Transjugular Intrahepatic Portosystemic shunt, INR, bilirubin, albumin and presence or severity of esophageal, gastric varices, ascites or hepatic encephalopathy. There were also no differences in baseline complication of liver disease (SBP, hepatorenal syndrome, hepatopulmonary syndrome, portopulmonary hypertension and hepatocellular carcinoma)

**Table S3** The cause of death in patients who died within 90days of initial evaluation in patients taking and not taking NSBB.

| **Cause of death** | **On NSBB** | **Not on NSBB** |
| --- | --- | --- |
| ***Liver related causes*** | | |
| Acute kidney injury | 1 | 1 |
| Gastrointestinal bleed MOF* | None | 1 |
| Sepsis MOF | 2 | 4 |
| Respiratory failure MOF | None | 1 |
| Retroperitoneal bleed MOF | None | 1 |
| MOF undefined precipitant | 1 | None |
| Portopulmonary hypertension (palliative care) | None | 1 |
| Unspecified ESLD (palliative care) | None | 1 |
| ***Non-liver related causes*** | | |
| Stroke | None | 2 |
| Metastatic cervical carcinoma | None | 1 |
| Unknown | None | 3 |
| Total | 4 | 16 |

Abbreviations: MOF, multiorgan failure; NSBB, non-selective beta blockers; ESLD end-stage liver disease

* Related to portal hypertension

**Table S4.** The association of NSBB use with 90-mortality in models using propensity score matching for use of NSBB at baseline,

| **Model** | **Coefficient** | **Standard error** | **95% Confidence Interval** | **P value** |
| --- | --- | --- | --- | --- |
| Matching on MELD, CPS, MAP, gender | -0.19 | 0.06 | -0.31 to -0.63 | .003 |
| Adding NSBB indication (large or previously bleeding esophageal or gastric varices) | -0.19 | 0.1 | -0.38 to 0.004 | .055 |
| Adding potential NSBB contraindication (refractory ascites or spontaneous bacterial peritonitis) | -0.27 | 0.09 | -0.44 to -0.08 | .004 |

Abbreviations. NSBB, non-selective beta blockers; MELD, model for end-stage liver disease; CPS, Child Pugh score; MAP, mean arterial pressure.

**Table S5.** The precipitating factors of acute kidney injury developing within 90 days of initial evaluation.

| **Precipitating factor** | **On NSBB**  **n=14** | **Not on NSBB**  **n=11** |
| --- | --- | --- |
| ***Prerenal related to infection*** | | |
| Clostridium difficile | 1 | None |
| Spontaneous bacterial peritonitis | 3 | None |
| Urinary tract infection | 2 | None |
| Sepsis-no defined source | 2 | None |
| ***Other prerenal causes*** | | |
| Prerenal, unspecified | 1 | 4 |
| Hepatorenal syndrome, unspecified | 2 | 2 |
| Gastrointestinal bleeding* | None | 1 |
| Dehydration | 1 | 2 |
| Hypotension | None | 1 |
| Retroperitoneal bleed | 1 | None |
| ***Unclassified*** | | |
| Intestinal ischemia | None | 1 |
| Unknown-outpatient | 1 | None |

Abbreviations: NSBB, non-selective beta blockers

* Related to portal hypertension

**Table S6** Comparison of selected characteristics and 90-day outcomes of patients with refractory ascites or a history of spontaneous bacterial peritonitis prior to initial evaluation for liver transplantation grouped by NSBB use. Values are shown as median (interquartile range) or number (percentage).

|  | **On NSBB**  **n=20** | **Not on NSBB**  **n=24** | **P value** |
| --- | --- | --- | --- |
| Age, years | 59 (52 - 64) | 58 (50 - 63) | NS |
| Male gender | 13 (65%) | 16 (67%) | NS |
| Heart rate beats/min | 65 (59 - 72) | 80 (70 - 97) | .002 |
| Mean arterial pressure, mmHg | 77 (71 - 85) | 84 (77 - 90) | NS |
| Child Pugh Score  Child Pugh class  A  B  C | 11 (10 - 13)  None  3 (15%)  17 (85%) | 10 (9 - 12)  None  8 (33%)  16 (77%) | NS  NS |
| MELD | 17 (15 - 21) | 18 (13 - 22) | NS |
| 90-day outcomes | | | |
| Acute kidney injury | 4 (20%) | 4 (17%) | NS |
| Gastrointestinal bleeding* | None | None |  |
| Spontaneous Bacterial Peritonitis | 3 (15%) | 1 (4%) | NS |
| Liver transplantation | None | 1 (4%) | NS |
| Mortality | 2 (10%) | 4 (17%) | NS |

Values shown as median (interquartile range) or number (percentage)

Abbreviations: MELD, Model for end-stage liver disease; NSBB, non-selective beta blockers

*Related to portal hypertension

**Table S7** The association of mean arterial pressure at the time of initial liver transplant evaluation with 90-day mortality and acute kidney injury using the area under the receiver operator characteristic curve, and sensitivity and specificity for a MAP<82mmHg to predict these endpoints. Thirteen (36%) of 36 patients not taking NSBB with a baseline MAP<82mmHg died within 90 days, as opposed to 3 (5%) of 63 patients with a higher MAP, p<0.001. Whereas, 3 (9%) of 32 patients taking NSBB with a MAP<82mmHg died within 90 days, compared with 1(3%) of 33 with a higher MAP, p=0.3.

| ***Endpoint of 90-day mortality*** | | | |
| --- | --- | --- | --- |
|  | **C-statistic (95% CI)** | **Sensitivity for MAP<82mmHg** | **Specificity for MAP<82mmHg** |
| All patients | 0.76 (0.7 - 0.82) | 84% | 64% |
| No NSBB | 0.81 (0.71 - 0.88) | 87% | 64% |
| NSBB | 0.65 (0.44 – 0.86) | 75% | 52% |
| ***Endpoint of 90-day acute kidney injury*** | | | |
|  | **C-statistic (95% CI)** | **Sensitivity for MAP<82mmHg** | **Specificity for MAP<82mmHg** |
| All patients | 0.70 (0.57 – 0.82) | 68% | 62% |
| No NSBB | 0.82 (0.66 – 0.98) | 82% | 62% |
| NSBB | 0.57 (0.39 – 0.74) | 57% | 53% |

Abbreviations: NSBB, non-selective beta blockers
